# Online Perspectives of Workers Navigating Workers’ Compensation Systems: A Content Analysis of the Reddit Social Media Platform

**DOI:** 10.7759/cureus.50733

**Published:** 2023-12-18

**Authors:** Zaira S Chaudhry, Crystal Widarma, Genevieve Saliuk

**Affiliations:** 1 Occupational Medicine, Loma Linda University Medical Center, Loma Linda, USA; 2 Occupational Medicine, University of Texas Medical Branch at Galveston, Galveston, USA

**Keywords:** patient education, reddit, social media, occupational injuries, workers’ compensation

## Abstract

Background: Social media platforms are increasingly used by the general public as a source of information on health-related and legal concerns, among other topics. Reddit.com, one of the top 10 most visited websites in the United States, is a popular social media platform that allows users to anonymously discuss various topics, including workers’ compensation (WC). Understanding the candid concerns of workers who are navigating WC systems will allow for the development of more effective educational resources that are tailored to the needs of this population.

Methods: In January-March 2023, a cross-sectional review of anonymous public posts submitted to the r/WorkersComp section of the Reddit social media website between December 2021 and December 2022 was performed. Post content was extracted from a systematic random sample and coded into themes/sub-themes and emotional tones by two independent reviewers. A third reviewer resolved any discrepancies in coding in order to reach consensus prior to data analysis. The data were analyzed using Microsoft Excel 2019 (Microsoft Corporation, Redmond, WA, USA).

Results: Content from 200 original posts submitted to r/WorkersComp was reviewed and analyzed. Nearly 94.0% of posts (n =187) specified a state of residence, with posters most frequently residing within the United States in California (32.0%), New York (7.0%), Pennsylvania (5.0%), and Florida (5.0%). The most common primary theme was “medical” (27.0%, n = 54), with questions and comments related to provider complaints, medical care access, referral denials, maximum medical improvement, and independent medical examinations being the most frequent within this category. The second most common primary theme was “legal” (26.5%, n = 53), with questions and comments related to lawyer retainment and settlements being the most frequent within this category. The third most common primary theme was “general” (18.5%, n = 37), with questions and comments related to the general claims process, eligibility for WC, claim denial, and communication issues with claims adjusters being the most frequent within this category. The fourth most common primary theme was “employer” (14.0%, n = 28), with questions and comments related to employer retaliation, job security, and work restrictions being most frequent within this category. Only 37.0% of posts (n = 74) expressed a clear emotional tone, with frustration (13.5%, n = 10), fear (13.5%, n = 10), and confusion (13.5%, n = 10) being the most frequent tones observed in this sample of posts.

Conclusions: Our findings indicate that there are workers who are navigating WC systems who use social media platforms such as Reddit to obtain information and advice on various aspects of WC, including medical issues, legal advice, and employer concerns. These findings may be used to address the information and education needs of workers who are navigating WC systems, which may help attenuate some of the frustrations surrounding the WC claims process.

## Introduction

Social media platforms are increasingly used by the general public as a source of information on health-related and legal concerns, among other topics. Reddit.com, one of the top 10 most visited websites in the United States, is a popular social media platform that allows users to anonymously discuss various topics, including workers’ compensation (WC). In an era where information is overwhelmingly obtained online, it is not surprising that individuals are utilizing social media as a means for obtaining information on medical and legal issues. Prior studies have reported on social media content related to various other medical specialties, such as dermatology, ophthalmology, and addiction medicine [1-3].

Buntinx-Krieg et al. explored dermatology-related content on the Reddit social media platform and found that most postings were related to seeking health/cosmetic advice [1]. Likewise, Mahjoub et al. examined Reddit content related to ophthalmology and reported that most postings were about diagnoses, surgical complications, and medication alternatives, with users who expressed emotional tones being most frequently perceived as anxious or worried. Of note, many of the contributors were self-identified optometrists and ophthalmologists [2]. Valdez et al. explored Reddit content related to addiction and recovery from substance use disorders and found robust dialogue among this online community; they concluded that such social media platforms may provide unique opportunities for building social connections among individuals with substance use disorders [3].

To the best of our knowledge, a social media analysis of the online perspectives of workers who are navigating WC systems has not been previously reported in the peer-reviewed literature. Given the complexities of WC and the various stakeholders involved, an examination of online anonymous dialogue on this topic may provide insights regarding the experience of the injured worker. Such social media content may be of particular interest to the various stakeholders in WC, as understanding the candid concerns of workers navigating WC systems may allow for the development and dissemination of effective educational resources tailored to this population's needs. Therefore, we sought to explore the candid concerns of workers who are navigating WC systems through a content analysis of the Reddit social media platform, as this particular platform lends itself to more robust text-based discussions without imposing character limits. This article was previously presented as a poster presentation at the 2023 American Occupational Health Conference on April 16, 2023.

## Materials and methods

The protocol utilized in the present study was adapted from prior studies in different medical specialties published by Buntinx-Krieg et al. [1], Mahjoub et al. [2], and Valdez and Patterson [3]. Institutional Review Board approval was not sought as this was a cross-sectional descriptive analysis of existing content published on a publicly available social media platform’s website by anonymous users. The Reddit social media platform allows individuals to create an anonymous account and post on a variety of topics, including medical and legal issues. User-generated content or postings are organized into various topic-specific sections known as “subreddits.” There is no restriction on who can access and view content posted on public subreddits. The Reddit platform is known for its "upvote" and "downvote" system, which allows users to express their approval or disapproval of a post or comment, thereby surfacing the most popular and engaging content at the top of the webpage, where it is most visible to users.

Between January 2023 and March 2023, a cross-sectional review of the publicly available posts on the WC subreddit of the Reddit social media platform, known as "r/WorkersComp," was performed. A systematic random sample of the most recent 1,000 original posts submitted to the r/WorkersComp subreddit within the preceding year (December 2021 to December 2022) was selected and included in this review. A preset sampling interval of five was used to select the systematic random sample; in other words, every 5th original post from a reverse chronological list of the most recent 1,000 original postings in the r/WorkersComp subreddit was included in this analysis.

Data extracted from each original post included title, text, date, username of the poster, state of residence of the poster (if disclosed), number of comments/replies per posting, and whether or not there was at least one comment/response from a “flaired” user. On the Reddit social media platform, a flaired user is a vetted user whose credentials have been verified by a Reddit forum moderator; this designation allows users to maintain anonymity within the Reddit community while providing credibility to their posted content.

Two independent reviewers collected the aforementioned data and coded original posts into various themes/sub-themes and emotional tones based on their content using a dynamic coding system within the data collection spreadsheet that allowed reviewers to manually add sub-themes and emotional tones as they encountered them during data collection. Primary themes represented broad topic areas, which included medical, legal, general, employer, and others (used for content falling outside the scope of the aforementioned content areas), whereas sub-themes represented specific questions/concerns within those broader themes. To avoid subjective interpretation of emotional tones, only emotions that were explicitly expressed were recorded (e.g., "I am so frustrated.").

Upon completion of data collection by both independent reviewers, a third reviewer reviewed each compiled dataset and resolved any discrepancies in coding in order to reach a consensus prior to the final data analysis. Data were compiled into a standardized spreadsheet and descriptive analysis [e.g., frequencies/percentages, arithmetic mean/standard deviation (SD)] of the reviewed content was performed in Microsoft Excel 2019 (Microsoft Corporation, Redmond, WA, USA).

## Results

**Figure 1:**
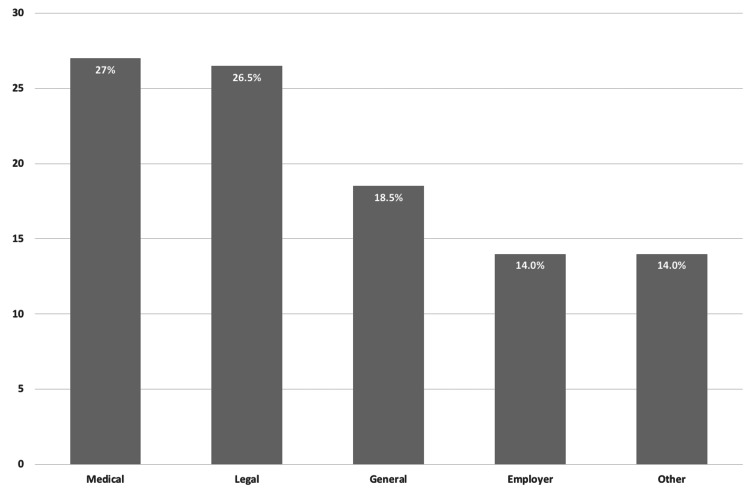
Distribution of primary themes of reviewed workers’ compensation Reddit posts.

The analysis included content from 200 posts submitted to r/WorkersComp between December 2021 and December 2022. Nearly 94.0% of posts (n =187) specified a state of residence, with posters most frequently residing within the United States in California (32.0%), New York (7.0%), Pennsylvania (5.0%), and Florida (5.0%). A wide range of themes were noted among the reviewed posts. Figure 1 illustrates the distribution of primary themes for the reviewed posts. The most common primary theme was “medical” (27.0%, n = 54), with questions and comments related to provider complaints, medical care access, referral denials, maximum medical improvement, and independent medical examinations being the most frequent within this category. The second most common primary theme was “legal” (26.5%, n = 53), with questions and comments related to lawyer retainment and settlements being the most frequent within this category. The third most common primary theme was “general” (18.5%, n = 37), with questions and comments related to the general claims process, eligibility for WC, claim denial, and communication issues with claims adjusters being the most frequent within this category. The fourth most common primary theme was “employer” (14.0%, n = 28), with questions and comments related to employer retaliation, job security, and work restrictions being most frequent within this category. The remainder of posts did not fall into any of the aforementioned categories and were classified as “other” (14.0%, n = 28), which included sub-themes such as the impact of quitting on an open WC claim, ability to attend school while on temporary disability due to a work-related injury, and financial concerns.

Only 37.0% of posts (n = 74) expressed a clear emotional tone, with frustration (13.5%), fear (13.5%), and confusion (13.5%) being the most frequent tones observed in this sample of posts. Table 1 delineates the frequency of each emotion expressed in this sample of postings. The mean number of comments or replies per post was 7.3 (SD = 7.2). Nearly 62.0% (n = 123) of posts had at least one comment or reply from a flaired/vetted user, with claims adjusters and attorneys being most represented among this group of contributors. Of note, none of the vetted users who contributed to this sample of posts were healthcare professionals.

**Table 1:** Emotions expressed by posters in sample of reviewed workers’ compensation Reddit posts.

Emotion	Number of posters (%)
Frustrated	10 (13.5%)
Fearful	10 (13.5%)
Confused	10 (13.5%)
Worried	9 (12.2%)
Stressed	6 (8.1%)
Curious	5 (6.8%)
Miserable	5 (6.8%)
Angry	4 (5.4%)
Distressed	3 (4.1%)
Skeptical	3 (4.1%)
Depressed	3 (4.1%)
Anxious	2 (2.7%)
Helpless	2 (2.7%)
Desperate	2 (2.7%)

## Discussion

To the best of our knowledge, a social media analysis of the online perspectives of workers who are navigating WC systems has not been previously reported in the peer-reviewed literature. Our findings indicate that some workers are actively engaging in online discussions via social media to seek information and advice on various aspects of WC, including medical issues, legal advice, and employer concerns. When these workers expressed emotions online, they were most often perceived to be frustrated, fearful, or confused. Although over half of the postings had at least one comment or reply from a “flaired” or vetted user with expertise in WC, given that state WC systems, regulations, and policies vary considerably throughout the U.S., such advice may not always translate across state lines. Needless to say, these findings highlight the importance of timely access to and availability of informational and educational resources in the WC claims process, as this can potentially help attenuate delays in care, miscommunications, and adversarial interactions that may ultimately prolong the recovery timeline for injured workers.

Our findings parallel prior research demonstrating that injured workers tend to lack basic knowledge regarding the WC claims process and often report poor claims administration as well as adversarial interactions as sources of frustration when surveyed about their experiences navigating WC systems [4,5]. Moreover, additional research has highlighted how such perceived adversarial interactions may lead to secondary psychosocial harm to injured workers [6,7]. While it was beyond the scope of the present study to determine what informational and educational resources these particular workers had been provided with or had access to, it is important to note that many state WC websites have robust resources available online to help workers navigate the claims process, including the top states represented among this particular sample of posters (California, New York, Pennsylvania, and Florida) [8-11]. Moreover, many major WC insurers also have informational resources on the claims process readily available online for claimants to access [12-14].

However, it is unclear if workers are aware of the aforementioned resources or if these resources are effective at educating workers on the WC claims process. In 2021, Vanderhooft demonstrated that 96.4% (n = 536) of surveyed WC claimants in the state of Utah lacked basic knowledge of the principles surrounding their claim, despite this information being available in the Utah Labor Commission Employee's Guide to Workers' Compensation and an employee information sheet; in fact, none of the survey participants were familiar with the employee information sheet [4]. These findings certainly raise the point that the mere existence of educational resources about the WC claims process does not ensure that the target population is being effectively educated.

Of course, this study is not without its limitations. Due to the qualitative nature of social media content, there is inherent subjectivity in social media content analysis. To minimize subjectivity in our content review process, our study design entailed data collection by two independent reviewers with the resolution of discordant data by a third reviewer to minimize misclassification bias. Next, this Reddit population represents only a subset of the population of interest and is not necessarily representative of all injured workers; in fact, prior demographic reports indicate that Reddit users tend to be younger, with a predominance of males, and have higher educational attainment than the general U.S. workforce [15]. It is important to note that WC systems within the U.S. are not uniform, and injured workers in different states may have distinct questions or concerns, which necessitates targeted approaches to WC education and information dissemination. Our findings are also not generalizable to other countries given global differences in healthcare and legal systems, as well as social media usage. However, we reviewed a large number of postings selected via systematic random sampling to capture a representative sample and minimize sampling bias within this subset of injured workers who post on the r/WorkersComp subreddit.

It is important to note that the r/WorkersComp subreddit is targeted toward workers who are seeking information; therefore, injured workers with positive experiences navigating WC systems may be less likely to share their experiences within this online community. Finally, ascertaining what educational/informational resources these injured workers had access to was beyond the scope of this study design. It is possible that some of the workers seeking information in this online setting may have had access to other educational/informational resources from their employers, state WC websites, and insurance carriers but were unaware that these contained the answers to their questions or simply wanted to obtain input/advice from external parties. In addition, Reddit and other social media platforms may provide an opportunity to seek support in an online community of other workers who are also navigating WC systems.

## Conclusions

Social media content provides insights into the candid concerns and information needs of workers who are navigating WC systems. Our findings indicate that there are workers who are navigating the WC system who use social media platforms such as Reddit to obtain information and advice on various aspects of WC, including medical issues, legal advice, and employer concerns. When these workers expressed emotions online, they were most often perceived to be frustrated, fearful, or confused. The results of this study indicate that there is a need for further research on this topic, including surveys and educational intervention studies, to gain a better understanding of effective strategies for educating injured workers on the WC claims process. Needless to say, given the variability of WC systems across the U.S., a targeted approach to WC education and information dissemination is warranted. By providing insights into the aspects of WC that appear to be a source of confusion for injured workers, these findings may be used to address the information and education needs of this population, which may help attenuate some of the frustrations surrounding the WC claims process.
